# Nurses' Experiences of Pain Management for Patients With Diagnosed Mental Health Conditions: A Systematic Review

**DOI:** 10.1111/jan.70626

**Published:** 2026-04-29

**Authors:** Aaron Lapuz Alejandro, Yvonne Middlewick, Seng Giap Marcus Ang, Rosemary Saunders, Irene Ngune

**Affiliations:** ^1^ School of Nursing and Midwifery Edith Cowan University Perth Western Australia Australia; ^2^ Centre for Research Into Aged Care EUC Perth Western Australia Australia; ^3^ Royal Perth Bentley Group Hospital Perth Australia

## Abstract

**Aim:**

To synthesise the available evidence related to nurses' pain assessment and management practices for patients with diagnosed mental health conditions.

**Design:**

Mixed‐methods systematic review.

**Data Sources:**

Medline, CINAHL, PsycINFO, SPORTDiscus and Psychology and Behavioural Sciences Collection.

**Methods:**

Databases search was conducted in March 2024 and updated in June 2025. Methodological quality was assessed using the Mixed Methods Appraisal Tool. Data synthesis followed the Joanna Briggs Institute (JBI) Manual for Evidence Synthesis.

**Results:**

Of the 1318 eligible studies identified, 12 met the inclusion criteria. The included studies were methodologically robust overall but frequently neglected nonresponse bias and were predominantly conducted in high‐income countries, potentially limiting generalisability. The synthesis revealed diverse experiences among nurses in assessing and managing pain in patients with diagnosed mental health conditions. Six key themes emerged: *Inadequate and Inconsistent Pain Assessments*, *Seeing is Believing*, *To Trust or Not to Trust the Patient*, *A Balancing Act*, *Diagnostic Overshadowing* and *Organisational Restraint and Support*.

**Conclusions:**

This review highlights the ongoing challenges nurses encounter in assessing and managing pain and the need for enhanced education and institutional support to strengthen nurses' capacity for effective pain assessment and management in patients with diagnosed mental health conditions. Stigma, both structural and interpersonal, continues to shape clinical decision‐making, often leading to under‐assessment and inadequate treatment of pain. Integrating clinical judgement with validated pain assessment tools will help ensure nurses provide evidence‐based pain management for this often‐marginalised group.

**Implications for Patient Care:**

Enhancing nursing competence in pain assessment and management for patients with diagnosed mental health conditions leads to more accurate and timely pain relief, significantly improving physical and psychological wellbeing. Effective pain control for this vulnerable group can reduce hospital length of stay and minimise complications, ultimately contributing to better health outcomes and quality of life.

**Patient Contribution:**

No patient contribution.

**Impact:**

*What problem did the study address?*: Pain is a complex sensation affecting people with diagnosed mental health conditions. They are likely to receive inadequate pain assessment and management due to mental health conditions preventing them from accurately self‐reporting their pain and advocating for timely treatment. This review explored the pain assessment and management practices among nurses for patients with diagnosed mental health conditions.

*What were the main findings?*: Significant barriers were identified, including inconsistent use of pain assessment tools, limited knowledge and confidence among nurses, power imbalances in the nurse–patient relationship, diagnostic overshadowing where physical symptoms are presumed to be related to mental illness, and organisational constraints such as staffing shortages, lack of education and restrictive hospital policies. Strategies to mitigate these barriers are essential to improving pain outcomes and promoting person‐centred care for this vulnerable population.

*Where and on whom will the research have an impact?*: This review highlights the need for more robust approaches to the assessment and management of pain experienced by patients with diagnosed mental health conditions. It underscores the importance of integrating pain assessment, mental health and substance use education into nursing curricula and post‐registration nursing practice. The findings highlight the need to update clinical guidelines and organisational policies, ensuring that nurses receive the necessary training, resources and support to provide effective pain management for patients with mental health conditions to enhance the quality of care and promote more equitable health outcomes for individuals with mental health conditions.

**Reporting Method:**

Joanna Briggs Institute (JBI) Manual for Evidence Synthesis and Preferred Reporting Items for Systematic and Meta‐analysis (PRISMA) reporting guideline for systematic reviews.

**Patient or Public Contribution:**

None.

## 1 Introduction

Pain is a common yet complex sensation affecting many people. In Australia, one in five Australians aged over 45 experienced chronic pain (Australian Institute of Health and Welfare 2020). Global estimates in 2022 indicated that approximately 27.5% of adults aged 25 and over experienced chronic pain, with prevalence rates ranging from 9.9% to 50.3% (Zimmer et al. 2022). While the experience of persistent pain is more likely to affect older people, people diagnosed with mental health conditions are also susceptible to experiencing both acute and chronic pain, although the prevalence of acute pain is rarely reported due to its evolving nature. Individuals, especially those with serious mental illness, tend to experience higher rates of physical health conditions, such as metabolic disorders, cardiovascular disease, obesity, cancer and respiratory illnesses, compared to the general population (Rotella et al. 2020). These physical health conditions, particularly cardiovascular and metabolic conditions, are strongly associated with persistent pain (Goodson et al. 2013), therefore placing them at a higher risk of hospitalisation and experiencing a reduced life expectancy (Onwumere et al. 2022; Sara et al. 2021).

Pain and mental illness are often interconnected and may contribute to or exacerbate each other (American Psychiatric Association 2020). According to Saunders' concept of ‘*Total Pain’* (1964), pain encompasses physical, psychological, social and spiritual dimensions, all of which influence the individual's overall experience of suffering (Mehta and Chan 2008). This multidimensional understanding helps explain why mental health disorders can intensify the perception of pain and why chronic pain can, in turn, worsen psychological distress. Moreover, due to the connected biological neural mechanism between mental health conditions and chronic pain, people with chronic pain are at risk of experiencing mental health issues, and those with mental illness are more sensitive to and at risk of suffering from chronic pain (Hooten 2016). A systematic review reported that patients with bipolar disorder are two times more likely to experience pain compared to the general population (Stubbs, Eggermont, et al. 2015). Another multinational population study also found depression to be associated with severe pain (Stubbs et al. 2017). Pain could have deleterious impact on the person's health and quality of life, which could in turn worsen the mental health condition. Therefore, it was suggested that proper assessment and treatment of pain is necessary as part of managing the psychiatric condition (Stubbs, Gardner‐Sood, et al. 2015). Adopting a *‘Total Pain’* approach encourages clinicians to address both the physical and psychological components of suffering to improve holistic well‐being (Mehta and Chan 2008).

Studies have reported that nearly half (46%) of individuals with serious mental illnesses, such as major depression and bipolar disorder, also have concurrent substance use disorders and experience greater pain intensity and chronicity (Davis et al. 2017; Merrill et al. 2012; Sullivan et al. 2006; Sunderland et al. 2025). The concomitant mental illnesses, substance use disorders and pain often complicate the treatment and management of pain among patients with diagnosed mental health conditions. These challenges include chronic pain symptoms overlapping with the mental illnesses and concurrent substance use disorders, leading to diagnostic overshadowing, a phenomenon where a person's physical health symptoms are mistakenly attributed to their existing mental illness (Hallyburton and Allison‐Jones 2023).

For individuals with mental illness including substance use disorder, pain is often undertreated due to health professionals' concerns about relapse, opioid withdrawal, tolerance, addictive potential and a lack of health professionals' confidence in managing complex cases (Bell et al. 2013; Sowicz et al. 2022). Additionally, mental illnesses, such as depression and schizophrenia, can also alter an individual's ability to process pain (Kim et al. 2022), leading to the potential of underreporting by the patients or misinterpretation of symptoms by healthcare providers. This raises the possibility that pain may be under recognised, inadequately managed, or underreported in this population. Also, because of the subjective experience of pain, clinicians are often heavily reliant on self‐reported pain assessment tools, which may be unreliable among mental health patients with cognitive impairment or those unable to self‐report pain during the acute stage of the mental illness (Boring et al. 2021; Twycross et al. 2015).

Identifying people with mental health conditions who have or are at risk of pain is important to reduce distress, poorer health outcomes and prolonged hospital stays. As in the general population, pain is associated with increased disability, poorer quality of life and even increased suicide risk in patients with severe mental illness (Racine 2018; Stubbs, Gardner‐Sood, et al. 2015). Addressing these challenges requires a multidisciplinary approach to improve pain assessment and management pathways and develop an integrated treatment for this vulnerable population (Onwumere et al. 2022).

Nurses, as members of the multidisciplinary team, are the main group providing direct care and are responsible for assessing and managing pain. Their constant presence and frequent interactions with patients allow them to promptly address concerns and escalate care as needed. Clinical decision making for pain management is also a core component of nursing practice. Tanner's Clinical Judgement Model (2006) provides a useful framework for understanding how nurses make and refine these clinical decisions through four interrelated processes: noticing, interpreting, responding and reflecting. Through this model, nurses can systematically recognise cues of pain (noticing), interpret their significance in the context of the patient's mental health condition (interpreting), implement appropriate interventions (responding) and evaluate the effectiveness of these actions to inform future care (reflecting) (Tanner 2006).

However, evidence shows that factors such as nurses' attitudes, knowledge and the effects of diagnostic overshadowing can create significant barriers, particularly in the assessment and management of pain in individuals with concurrent serious mental illness and substance use disorders (Dewar et al. 2009). Goffman's (1963) *‘Stigma Theory’* further explains how negative societal and professional attitudes toward mental illness can lead to stereotyping, prejudice and discriminatory behaviours, all of which contribute to unequal care experiences (Goffman 2009). Such stigma may influence healthcare professionals' judgements, leading to the minimisation or misinterpretation of pain symptoms in patients with diagnosed mental health conditions (Bean et al. 2022). Additional barriers include the inadequate use of available tools for pain assessment or lack of consistent clinical standards for pain management in patients with cognitive impairment, serious mental illness and substance use disorders (Barney et al. 2020).

In the United States, the Joint Commission introduced a standard update of behavioural health care to introduce the new pain assessment and management requirements (The Joint Commission 2018). These standards emphasised the importance of providing appropriate screening, assessment and treatment of physical pain for all patients, especially those with mental, behavioural and substance use disorders. Pain frequently occurs together with mental and substance use disorders and must be co‐managed to achieve progress in treatment for the affected individuals. Despite the existence of guidelines, inconsistencies in the assessment and management of pain remain common for individuals with mental illness and co‐occurring substance use disorders (Callister et al. 2022). In Australia, there is no specific standard for pain management for patients with diagnosed mental health conditions. Instead, these practices are primarily guided by the National Standards for Mental Health Services (Australian Government 2010), and the Opioid Analgesic Stewardship in Acute Pain Clinical Care Standard (Australian Commission on Safety and Quality in Health Care 2022), which emphasises a bio‐psycho‐social approach to pain assessment.

To advance integrated approaches to pain management for patients with mental illness, it is essential to deepen our understanding of healthcare professionals, particularly nurses and their knowledge and practices in assessing and managing pain among patients diagnosed with mental health conditions including substance use disorders. A preliminary search of the literature revealed no synthesised review of pain assessment and management across these diagnostic groups that may help identify barriers to and facilitators of integrating existing and novel treatments for pain and mental illnesses in practice.

## 2 Aim

The aim of this mixed‐methods systematic review was to synthesise available evidence related to nurses' pain assessment and management practices for patients with diagnosed mental health conditions including substance use disorders.

## 3 Methods

### 3.1 Design

This mixed‐methods systematic review was guided by the Joanna Briggs Institute (JBI) Manual for Evidence Synthesis (Lizarondo et al. 2020) and the 2020 updated Preferred Reporting Items for Systematic and Meta‐analysis (PRISMA) statement (Page et al. 2021). This method combines both quantitative and qualitative data from primary studies to create a breadth and depth of understanding pain assessment and pain management for patients with diagnosed mental health conditions and to answer the review question ‘How do nurses assess and manage pain for patients with diagnosed mental health conditions?’ The protocol for the systematic review was developed in collaboration between all authors and registered with the International Prospective Register of Systematic Reviews (PROSPERO registration number: CRD420251082122).

### 3.2 Search Method and Strategy

A comprehensive search was conducted using MEDLINE, CINAHL, PsycINFO, SPORTDiscus and Psychology and Behavioural Sciences Collection in March 2024 and updated in June 2025. No date restrictions were applied. The search strategy used keywords and MeSH terms related to the three concepts: ‘nurses’, ‘pain’ and ‘mental disorder(s)’. An initial search was developed using Medline and validated with the University librarian. After which, the search terms were used for searching other databases. Additional records were also identified from the reference lists of all selected studies. The full search term list is presented in Table 1.

**TABLE 1:** Search strategy.

No	Terms	MEDLINE	CINAHL	PsycINFO	SPORTDiscus	Psychology and Behavioural Sciences Collection
1	Nurs*	1,178,702	1,004,162	211,602	17,887	42,420
2	MH “Nurses”	47,035				
3	Nurs* assistant*	15,793	13,272	2,244	242	605
4	MH “Nursing Assistants”	4,426				
5	Pain*	1,056,994	383,136	149,541	80,713	35,413
6	MH “Pain*”	158,282				
7	Pain Measur*	117,507	63,714	22,942	7,827	2,464
8	MH “Pain Measurement”	95,082				
9	Pain*assess*	50,233	24,589	11,350	3,999	1,799
10	Mental disorder*	252,632	91,863	217,679	2,645	15,805
11	Mental illness*	50,294	28,707	62,357	5,138	31,596
12	Psychiatric disorder*	69,478	17,075	60,611	1,690	10,103
13	Psychiatric illness*	13,276	3,882	12,627	260	1,975
14	MH “Mental Disorders*”	179,849				
15	1 or 2 or 3 or 4	1,178,702	1,004,162	211,602	17,887	42,420
16	5 or 6 or 7 or 8 or 9	1,056,994	383,136	149,541	80,713	35,413
17	10 or 11 or 12 or 13 or 14	327,812	116,587	277,175	7,998	47,781
18	15 AND 16 AND 17	607	255	357	30	69

### 3.3 Inclusion and Exclusion Criteria

To address the review question, the review included: (1) both qualitative and quantitative studies investigating nurses' knowledge and practices of pain assessment and management, (2) patients aged 18 years and over, with a mental health diagnosis in hospital and (3) published in English till June 2024. The definition of a mental illness/disorder followed the diagnostic criteria and definition in ICD10/11 and classifies substance use disorders within the chapter mental and behavioural disorders (F00‐F99) (World Health Organization 2019/2021). Hence, this review considered substance use disorders to be a standalone mental health condition. Pain assessment practices included: (1) physical pain being assessed by nurses using a valid tool, (2) clinical records about pain being reviewed, or (3) the record of clinician judgement about pain in patients with mental illness. The studies were excluded if they: (1) focused on children below 18 years old, (2) did not examine pain assessment or management practices among nurses, or (3) people with mental illness were not a target group. In addition, the studies that investigate the assessment of pain during an adverse event of a drug trial or as part of an experiment, or mental pain are excluded.

### 3.4 Selection Process

Following the search, all identified citations were entered into EndNote 2025 (Clarivate, PA, USA) and duplicates were removed. Titles and abstracts were screened by two independent reviewers (IN and AA) for assessment against the inclusion criteria for the review. Potentially relevant studies were retrieved in full and their citation details imported into Covidence (Veritas Health Innovation, Vic, Australia). The full text of selected citations was assessed in detail against the inclusion criteria by two independent reviewers (IN and AA). Any disagreements that arose between the reviewers at each stage of the study selection process were resolved through discussion with a third reviewer (YM). The results of the search are presented using the Preferred Reporting Items for Systematic Reviews and Meta‐analyses (PRISMA) flow diagram (Page et al. 2021, see figure 1).

### 3.5 Quality Appraisal

The Mixed Methods Appraisal Tool (MMAT) was used to appraise the quality of included studies (Hong et al. 2018). The critical appraisal was conducted by two independent reviewers (IN and MA), and any disagreement between the reviewers was discussed and resolved with the third reviewer (YM). The MMAT contains two screening questions: clarity of research questions and appropriateness of data collection method, and five criteria based on the study design. The criteria for qualitative and quantitative descriptive designs were used. Each reviewer rated each criterion using ‘yes’, ‘no’ or ‘can't tell’ and the overall quality for the included studies were discussed narratively. The critical appraisal of the included studies is presented in Table 2.

**TABLE 2:** Critical appraisal of included studies.

Study	Type	1.1. Qual approach appropriate	1.2. Data collection adequate	1.3. Findings derived from data	1.4. Interpretation substantiated	1.5. Coherence of components	4.1. Sampling strategy relevant	4.2. Sample representative	4.3. Measurements appropriate	4.4. Low nonresponse bias	4.5. Statistical analysis appropriate
Bohm et al. (2019)	Qualitative	▲	▲	▲	▲	▲	n/a	n/a	n/a	n/a	n/a
Dewar and Osborne (2004)	Qualitative	▲	▲	▲	▲	▲	n/a	n/a	n/a	n/a	n/a
Dewar et al. (2009)	Qualitative	▲	▲	▲	▲	▲	n/a	n/a	n/a	n/a	n/a
Kerner et al. (2013)	Quantitative descriptive	n/a	n/a	n/a	n/a	n/a	▲	▲	▲	▼	▲
Li et al. (2012)	Quantitative descriptive	n/a	n/a	n/a	n/a	n/a	▲	▲	▲	▼	▲
Morgan (2014)	Qualitative	▲	▲	▲	▲	▲	n/a	n/a	n/a	n/a	n/a
Morgan (2016)	Qualitative	▲	▲	▲	▲	▲	n/a	n/a	n/a	n/a	n/a
Morley et al. (2015)	Qualitative	▲	▲	▲	▲	▲	n/a	n/a	n/a	n/a	n/a
Morley et al. (2020)	Qualitative	▲	▲	▲	▲	▲	n/a	n/a	n/a	n/a	n/a
Rowe (2018)	Qualitative	▲	▲	▲	▲	▲	n/a	n/a	n/a	n/a	n/a
Riquin et al. (2017)	Quantitative descriptive	n/a	n/a	n/a	n/a	n/a	▲	▲	▲	▼	▲
Sullivan et al. (2015)	Quantitative descriptive	n/a	n/a	n/a	n/a	n/a	▲	▲	▲	▼	▲

*Note:* Black triangle—the criterion was assessed as being met; inverted grey triangle—the criterion was not met.

Abbreviation: n/a, not applicable.

### 3.6 Data Extraction and Outcomes

To ensure inter‐rater reliability, data were extracted independently by two reviewers (IN and AA) using a standardised data extraction form adapted from the JBI data extraction template in Microsoft Excel. The use of independent extraction by two reviewers allowed for cross‐checking of the extracted data to enhance consistency and reduce potential bias. Any disagreements between reviewers were discussed and resolved through consensus, with a third reviewer (YM) consulted where necessary. The data extracted from both quantitative and qualitative studies included details about the author, year and country, study aims, design, population (nurses and patients if applicable), setting and method (Table 3).

**TABLE 3:** Data extraction.

Author (year), country	Aims	Design	Nurse population	Sample	Patients	Setting	Method
Bohm et al. (2019), Sweden	To describe pre‐hospital emergency nurses' experiences of relieving pain in patients with substance abuse syndrome	Descriptive qualitative	Pre‐hospital emergency nurses/RNs with specialist ambulance nurse education	8 pre‐hospital emergency nurses active in the EMS setting for at least 2 years	Patients with substance abuse syndrome (addiction problems) requiring pain relief	Ambulance station	Semi‐structured interviews
Dewar and Osborne (2004), Canada Citation search	To explore the ways in which nurses in acute psychiatric settings assess pain and make decisions about pain management	Descriptive qualitative	Acute care psychiatric nurses RN/registered psychiatric nurses	5 RN/registered psychiatric nurses with 2 years minimum experience caring for patients in psychiatric setting	Patients in psychiatric settings	Acute care psychiatric unit	Semi‐structured interviews
Dewar et al. (2009), Canada Citation search	To explore the ways in which nurses in acute psychiatric settings assess pain and make decisions about pain management	Descriptive qualitative	RN or registered psychiatric nurses working in psychiatrics units	17 females and 3 males aged between 25 and 64 years with 3.5 to 40 years of nursing experience	Patients in psychiatric settings	6 acute care psychiatric units in 3 urban areas of Western Canadian province	Semi‐structured interviews
Kerner et al. (2013), Israel Citation search	To assess the performance of pain evaluation and management by nurses in patients admitted in internal medicine wards and to identify groups of patients (including mental disorders as a cause of hospitalisation) in which pain evaluation was insufficient	Observation retrospective study		59 patients	Patients who were admitted in one of six internal medicine wards	Internal medicine wards	Medical records review
Li et al. (2012), Norway	To describe health professionals' interventions, knowledge, and attitude towards opioid‐dependent patients with pain	Descriptive cross‐sectional	Nurses and doctors working at 4 Norwegian hospitals in 4 different regions	274 nurses and 38 doctors completed questionnaire	patients with substance abuse and pain	6 medical, 3 surgical and 3 intensive care or anesthesia wards.	36 questions covering background variables, actions, knowledge, and attitudes
Morgan (2014), United States	To expand the knowledge about nurses' attitudes and interactions with patients with substance use disorders who were in pain	Grounded theory	Nurses who worked with patients with substance use disorder who were in pain	12 females and 2 males aged between 31 and 61 years old	Patients with substance use disorders	Urban public health hospital	Semi‐structured interviews
Morgan (2016), United States	To explore both hospice/palliative care nurses' and psychiatric/mental health nurses' attitudes and needs toward people with severe mental illness at the end‐of‐life	Qualitative phenomenology	Hospice/palliative care and psychiatric nurses who has experienced in looking after patients with severe mental illness during end‐of‐life care	18 females and 2 males including 6 = psychiatric/mental health; 7 = hospice/palliative care and 2 = both disciplines; 5 = others	Patients with serious mental illness at the end‐of‐life	Psychiatric, hospice or palliative care unit	Semi‐structured interviews
Morley et al. (2015), United Kingdom	To gain a deeper understanding of the experiences of nurses and the barriers between them and patients with substance use disorder experiencing pain	Qualitative phenomenology	RN with experience in emergency, vascular, education, trauma & orthopaedics, prison service, palliative care, pain services and accident & emergency	5 RNs	Patients with substance use disorder	NA	Semi‐structured interviews
Morley et al. (2020), United Kingdom	(1) To explore the ethical issues from nurses' experiences of working with patients with SD in pain, based on the four principles of biomedical ethics; (2) To identify ethical tensions and the implicit ethical analysis nurses employed when faced with ethical challenges encountered in clinical practice; (3) To make normative recommendations for clinical practice	Qualitative phenomenology	Post‐registration nurses of clinical backgrounds including ED, vascular, education, trauma & orthopedics, prison service, palliative care, pain services, and accident & emergency	5 RN/registered psychiatric nurses with 2 years minimum experience caring for patients in psychiatric settings	Patients with substance‐use disorder (SD)	NA	Secondary analysis of themes generated in during semi‐structured interviews
Rowe (2018), United States Citation search	To understand psychiatric nurses' attitudes, personal observations, and experiences in working with psychiatric patients diagnosed with a chronic pain disorder, and its influence on the delivery of patient care within a long‐term care psychiatric hospital	Qualitative phenomenology	RNs in psychiatric unit	4 females and 1 male aged between 29 and 64 years old	Long‐term psychiatric patients with chronic pain	Long‐term psychiatric unit	Semi‐structured interviews
Riquin et al. (2017), France	To illustrate personal observations, and experiences in working with psychiatric patients diagnosed	Case study	NA	1 RN in psychiatric unit	Patients with schizophrenia during end‐of‐life care	Psychiatric unit	Clinical vignette
Sullivan et al. (2015), United States Citation search	To examine the effect of a mental health diagnosis (schizophrenia) on providers' clinical expectations and decision making regarding physical health care	Cross‐sectional survey	Primary care and mental health nurses and physicians	275 providers (sample size for nurses not specified)	Patients living with schizophrenia	5 US Veteran Affairs medical centres	Video vignette case studies with a 26 items survey

### 3.7 Data Transformation and Synthesis

For the quantitative studies, it was not possible to conduct a meta‐analysis because the included studies varied greatly from each other in terms of design, methods and populations. Following extraction, the quantitative data were converted into ‘qualitised data’, which involved transforming them into textual descriptions or narrative interpretations to respond directly to the review question. In line with the JBI methodology for mixed methods systematic reviews, a convergent integrated approach was adopted (Lizarondo et al. 2020). The qualitised data were assembled with the qualitative data by categorising and pooling them based on similarity in meaning to produce a set of integrated findings. The thematic synthesis approach was chosen to enable the identification of recurring patterns and overarching themes across the heterogeneous evidence base, allowing for a comprehensive interpretation that could not be achieved through statistical pooling. The data synthesis and classification of pain assessment and management are presented in Table 4.

**TABLE 4:** Data synthesis.

No.	Author (year), country	Themes from article	Supporting evidence	Theme
1.	Bohm et al. (2019), Sweden	Pre‐emergency nurses (PENs) often found it difficult to trust pain reports from patients with substance use histories when no visible injuries or abnormal signs were present, leading to restrictive pain management. In contrast, patients with clear physical injuries were readily believed and received prompt, adequate pain relief	I go very much on body language and parameters, I mean they're sitting there and talking fluently and saying they have VAS 10 on the pain scale, but they have normal blood pressure and normal pulse and no dilated pupils, so there is nothing physiologically that indicates that they're in pain at all.	Invisibility of pain
		All PENs commented that they wanted to believe patients who complained of pain, pointing out that people who were injured had a right to treatment	I assess them like all other patients, and I think that's what one should do. I think no matter what we know or think we know, with patients complaining of pain, we should alleviate [their pain] if we can.	Trusting the patient
		While providing pain relief medication was a key aspect of care, PENs also emphasised the importance of building trust, offering reassurance, and creating a calm, supportive environment. These additional measures were seen as essential to delivering ethical, high‐quality, and person‐centred pain management	I can listen to the patient and, like, try to calm the patient down and give the patient time and just be there, so to speak. It may relieve the pain and maybe the situation will be different after a while, but otherwise when you say pain relief, I think of the medications we have. First and foremost	Holistic caring
		In cases involving known substance abuse, PENs often chose to be more restrictive with pain medications, sometimes experimenting with alternative treatments	We've ended up in situations where we meet people who, well, fake an emergency just to get hold of drugs. Of course, they never get given drugs.	Withholding treatment
		When patients specifically requested certain analgesics and dosages, PENs often interpreted this as a sign of possible drug‐seeking behaviour. This perception influenced their level of trust and approach to pain management	But it doesn't feel right, going and giving him as high doses as he wants, in this case morphine and then leaving, that's not the way we usually work, it doesn't feel OK, you feel frustrated and that isn't any good for the patient or for us.	Feelings of manipulation
		In situations of doubt or uncertainty around pain relief, PENs frequently sought guidance from colleagues or other professionals. This support helped them feel more confident and less isolated in their decision‐making, ultimately improving their ability to manage patients pain	Call a doctor and get support from the ED and ask there. I can describe the patient's problems and how I feel, so if they (ED) can pick up anything on him (previous journal notes), get a prescription from there, a direct order, affects what I, how I should relate to the patient.	Organisational facilitator
2.	Dewar and Osborne (2004), Canada Citation search	Their pain (patients with MH problems) is viewed and treated differently than it would be if they were medical or surgical patients		Invisibility of pain
		The nurses reported the dilemma of trying to relieve pain yet provide care that would not contribute to addictive behaviours		Withholding treatment
		Patients with mental health problems often carry the stigma of mental illness into all healthcare settings, resulting in their pain being less prioritised. In emergency departments, once a psychiatric 'label' is assigned, pain assessment becomes secondary, frequently leading to inadequate treatment, prolonged admissions, and greater patient distress		Diagnostic overshadowing
3.	Dewar et al. (2009), Canada Citation search	When patients subtly pressure nurses for medication, it can evoke a strong sense of being manipulated, threatening the nurse's professional integrity and self‐worth. This perceived manipulation not only compromises their confidence in delivering effective care but also deeply challenges their identity as compassionate, ethical caregivers	It was like frustration, I was angry at myself for thinking, like, how did this happen, how did I get conned when you do your assessments and you try to be so thorough and that you're taking the best interest of your patient? As a nurse it just makes me feel that your credibility, not your credibility but your professionalism you could be the street drug corner guy giving out drugs to this guy, sometimes you feel like a pusher, you feel like the drug dealer and it makes you feel kind of yucky when you've got this sort of shady guy manipulating you and it leaves you feeling dirty afterwards.	Feelings of manipulation
		Nurses reported a clear dilemma when caring for patients with a history of addiction, expressing concern that prescribing opioids could trigger relapse or dependency. As a result, they often preferred to explore non‐opioid medications or alternative pain management strategies to balance effective care with harm reduction	If she rates her pain as high on a scale of 1 to 10, I would still give it to her. I would believe her but just having a previous addiction, it would still make me skeptical.	Withholding treatment
		Nurses has no issue administering pain medications if there is verifiable source (physical). However, if there is no known physiological source and if patient has history of drug addiction, administration of pain relief is not a resolution but a question mark	When they've been on a surgical floor and they've had a pin put in their leg and they ask me for Tylenol 3's for that leg pain. I mean the person has obvious pain when you're here [psychiatry] it's a little less obvious	Invisibility of pain
		Nurses in believed that in order to be seen as credible by physicians, they needed to present information in an objective, calm, and unemotional manner. Rational discussions of care helped foster agreement on treatment options, while emotional responses from nurses made it harder to reach consensus on pain management, ultimately making it more challenging for patients to receive pain medication	He [physician] respected the staff person [nurse] who was telling him. It's also how the staff person conveys the information. If they're objective and factual or if they're emotionally reactive, you get a very different response.	Organisational barrier
		Maintaining a calm environment is an important skill and exercising this skill contributes to being viewed as a good team member. Units managed by knowledgeable and experienced nurses had a reassuring, therapeutic, and healing atmosphere	It's the ones [nurses] that they [physicians] trust their judgment. When you walk onto the unit and certain nurses are on, you just feel everything is calm everything is being handled and everything goes fine. Other times you go on and it depends on who the nurse if they're highly anxious or they lack confidence in their own knowledge and skills or they just simply haven't had enough experience. You really feel it with the patients.	Organisational facilitator
		Nurse‐to‐nurse power dynamics significantly influence how patients are perceived and treated for pain. Nurses often anticipate their colleagues' reactions to the appropriateness of a decision, which in turn shapes their own choices regarding pain management	I medicated the patient, not because I wanted to medicate them, but because I knew my colleague would report me if I didn't and report me for not managing my patient properly. Well you can have the odd type of conversation where one nurse will say, Oh, it's all in his head but mostly we're pretty respectful of the patient.	Organisational barrier
		Nurses' beliefs about pain strongly impact their approach to pain management and their decision to administer analgesics. They often view pain holistically, considering physical, emotional, and psychological factors in their care decisions	I don't think you can separate pain in your psyche if you're undergoing chronic pain your psyche is constantly dealing with it and everything becomes harder, your nerves are frazzled, you're impatient, irritable, everything is more difficult.	Holistic caring
		Nurses' perceptions of addiction influence their decisions on administering pain medication, with some fearing that giving opioids may worsen the patient's addiction. This concern is often compounded by emotional and psychological reactions linked to societal stigma and judgment surrounding addiction	That was a fear (giving pain medication), that could start it all over again [patient's addiction] but yet the nurse in me was still saying, no, she deserves to be comfortable and we need to help her with that.	Withholding treatment
4.	Kerner et al. (2013), Israel Citation search	Patients with psychiatric diagnoses had lower odds of receiving appropriate pain evaluations, suggesting that once psychiatric issues were identified, pain assessment was not prioritized	Reduced chance of pain evaluation for mental illnesses vs. all others: OR 0.81, 95% CI 0.71–0.94, *p* = 0.004. Only 33.3% of the recommended pain assessment was done for patients with mental illness as the main reason for admission, compared to 58.1%–72.1% for those admitted for medical reasons.	Diagnostic overshadowing
5.	Li et al. (2012), Norway	Nurses exhibited hesitation in trusting pain reports from patients with substance use disorders, highlighting the influence of mental health problems influence pain management decisions	21% of nurses agreed or somewhat agreed with statement that opioid addicts tell the truth when they covey pain.	Invisibility of pain
			56% of nurses often felt that opioid drug addicts manipulated patients dependent on opioids. 29% of nurses claimed that opioid‐dependent patients tried to trick them when they asked for painkillers.	Feelings of manipulation
			< 50% administered opioid substitution to replace patients' daily use of opioids.	Withholding treatment
		Inadequate pain assessment	25% of respondents used pain assessment tool ‘very often’ and quite ‘often’.	Organisational barrier
6.	Morgan (2014), USA	Nurses' attitudes toward pain and addiction were influenced by various factors, including their cultural beliefs, professional experience, education level, and personal or familial encounters with pain and substance use	They think that the patient has a drug addiction. They worry that it's [opiates] going to affect them. They don't want to encourage addiction, so they don't want to give it. When they [nurses] think that somebody is addicted, they tend to overlook their pain. They think that those addicted patients have no pain. They're making it up and that's why they don't want to give it.	Withholding treatment
			Sometimes we say [to another nurse] ‘Why do you think that [that patient is a drug‐seeker]?’ ‘Oh, because he's a clock watcher. Oh, because, you know, he would ask for pain meds and the next thing you know, he's off the floor. So I don't think he's in pain’	Invisibility of pain
			They get very upset if you want to even approach them with something different. They just want the pill and they want it now. Or they want their injection or they want the needle but they want it now. In my experience they usually don't even want to hear any other suggestion and they get very upset: ‘My doctor ordered this for pain, my doctor said that he wanted me to have this’. Almost like you're trying to prevent them from getting the pills. They're very demanding, very demanding people; very difficult to please.	Feelings of manipulation
			Believe me, I'm trying to find ways because it is hard to give good care and when you have this negative feeling, it's hard to give good care, very hard, so I try ways to justify their behaviors so I can really work with them.	Holistic caring
			I'm advocating for the patient even though I know that the patient is taking a lot [of pain medication]. But at the same time I know that his tolerance is very, very high. Even though, you know, I'm the one asking for more [pain medication from the doctor] because I really and truly believe that the patient needs more or is it just because I want to get him out of my face? It's not the right thing to do to give him more just to shut him up.	Advocating
			The relationship between patients and nurses is, you know, is very important, and if you think about it, they've left their homes, or if they have homes or not, they're here, and to me nursing, like I tell other nurses, somebody's life is in your hands. They are trusting you and their life is in your hands. So if you don't look at pain, and then you don't treat them, you know. It's like you are contributing to their condemnation or whatever way you put it. So long as the person is here and we've taken a vow to care for the patient, I think we should do it with all our heart.	Trusting the patient
		Nurses identified significant workplace barriers that hindered their ability to provide effective pain treatment. These included low staffing levels, high patient acuity, difficulty reaching prescribers, documentation challenges, and a lack of resources, including access to other providers who could offer alternative pain management strategies	And you have, I'll say 14 patients for example, so the medication nurse is thinking about finishing her meds and yes, she still has to document, so right there it's already, Oh my God, will I ever finish? So there's really no time to enjoy the patient, or listening to ‘what really is your pain?’ and, I, you know, it's the computer, I think, is pulling us from our bedside nursing, it's preventing us from being at the bedside.	Organisational barrier
7.	Morgan (2016), USA	Nurses in this study highlighted the importance of maintaining a positive attitude towards psychiatric/mental health and palliative care nursing to ensure the best care for individuals with serious mental illness (SMI) at the end of life	I didn't take a strong enough stance with the (medical) providers who didn't want her to get methadone. It is something I always regretted and really learned from	Holistic caring
		Nurses expressed concern that the symptoms of serious mental illness interfere with their ability to provide quality care, including accurately assessing and managing pain	With the person who's hostile and paranoid and who not only has their mental illness to deal with but now they have a physical illness so now they're feeling things that are real and yet often unable to describe, unable to um, verbalize so sometimes the behaviors come out, people look at the behaviour, and the patient may be in pain but you don't, you're not clear about that, so that assessment is more complicated.	Invisibility of pain
		Nurses may experience concerns when caring for individuals with serious mental illness, especially regarding how psychiatric symptoms could impact the development of trust and rapport in new nurse‐patient relationships	And I was afraid that she would not be comforted by me or feel safe with me. I was really grateful and sort of a little bit overwhelmed by how nicely she accepted me. I felt she was more trusting than I would have expected somebody with a serious mental illness to be.	Trusting the patient
		Nurses described their need for support when dealing with patients with serious mental health illness in end‐of‐life‐care	I think they have to look at it and have an integrated program of not only serious mental illness, but also weave in addiction because the two in my opinion, it can't be one without the other. I think there is also a need to educate people in dealing with pain management and that number one, just because narcotics are prescribed you're not going to make everyone an addict.	Organisational facilitator
8.	Morley et al. (2015), UK	Nurses felt that decision‐making regarding pain management was largely controlled by doctors, limiting their ability to contribute. They also perceived that some doctors prioritised managing patients' addiction over ensuring adequate pain relief, creating tension in the delivery of care	Sometimes that is part of our role as well, to go and just outline to people that patient doesn't fit that mold, it is nobody's fault he is here and we have to try and manage that and work with that as best we can. I remember one of the juniors [doctors] in particular said, 'I am so glad you have said that. our consultant just thinks we can get him fixed' and I said yes, well they will, that is their mindset but this is our reality.	Diagnostic overshadowing
		Nurses frequently described patients with substance use disorders (SUD) in pain as ‘difficult’. These patients were often perceived as manipulative, aggressive, noncompliant, and distrustful, which contributed to overall challenging care situations	He could be a bit noncompliant, let's say, with his care, so things like if they were going to try and get him to have a wash he wouldn't be interested at all, always demanding more and more and more drugs, more and more painkillers, so he was tricky.	Feelings of manipulation
		Some nurses perceived doctors as unsympathetic toward patients with substance use disorders, particularly when their behaviours were seen as disruptive or non‐compliant. Nurses often felt their concerns about ongoing pain were dismissed, reflecting a lack of shared decision‐making and compassion in care	The doctors have got targets they have got to meet and an IV user comes in and sits in a bed for weeks on end with wound breaking down, keeps disappearing off the ward, keeps taking their dressings off themselves and things like this and they are just very unsympathetic and don't really want to talk about it if you say, well, they are still having a lot of pain, I think the doctor would probably say and.	Advocating
		Participants acknowledged that patients with substance use disorders (SUD) often have complex social and psychological needs, requiring a holistic, person‐centred approach. Recognising these factors, particularly when pain is involved, was seen as essential for providing compassionate, flexible, and collaborative care		Holistic caring
		Nurses believed that clinical experience with patients experiencing pain and substance use disorders (SUD) enhanced their ability to manage these patients more effectively	Experience is quite valuable in acquiring the confidence to give them quite high doses of opiate drugs because some people, particularly junior members of staff, feel quite uncomfortable about that.	Organisational facilitator
		Nurses believed that financial pressures on hospitals had strained resources, resulting in a lack of protected time for teaching and professional development. They also felt that policies focused on avoiding hospitalisation and promoting quick recovery contributed to doctors' attitudes of simply ‘fixing’ patients		Organisational barrier
9.	Morley et al. (2020), UK	Nurses sought to trust their patients, but many struggled to maintain neutrality, especially when patients had a history of substance use or displayed challenging behaviours	If people build up such tolerance over a period of time their normal is not like someone else's normal so you still have to give them appropriate analgesia and I think it is completely safe and right to do that	Trusting the patient
		Nurses described paternalistic attitudes among clinicians, where patients were often treated as though they lacked the capacity to make decisions about their own care. These behaviours frequently aligned with the medical model, which prioritises ‘fixing’ problems, sometimes undermining patient autonomy and neglecting individual needs	I had one doctor who said oh we can't give him opioids because he is meant to be weaning so that he only gave him 5 milligrams of Oramorph and he had a fractured pelvis, fractured limbs and a clavicle fracture but our doctor seemed obsessed with weaning and fixing the drug abuse problem.	Diagnostic overshadowing
		Nurses expressed reluctance to give patients full control over pain medication, especially those with substance dependence (SD), due to concerns about misuse. They described the need to ‘harden’ themselves and remain constantly vigilant, emphasising the emotional and ethical tension between effectively managing pain and preventing maladaptive behaviours	So there is always dread when the doctor says to me or the nurse handing over to me says this is potential drug use behaviour or they have substance misuse and there is pain there because basically you have to harden yourself. Any other patient you wouldn't hesitate to give them analgesia, you wouldn't hesitate to give them Morphine if that is what is going to make them comfortable.	Withholding treatment
			He wouldn't let them (take bloods) so there was a really weird deal that they would let him take his Oramorph if he would let them take bloods, so that was the compromise that was reached.	Feelings of manipulation
		Nurses described situations where patients with substance dependence (SD) were administered lower‐than‐usual doses of pain medication, even in cases involving extensive injuries	I don't know how to phrase this in a way that is appropriate, but I think there is always that worry that, perhaps giving opiates to somebody who is addicted to opiates is not a good idea but sometimes that is what they need.	Withholding treatment
		Nurses felt a strong sense of responsibility to advocate for their patients' pain relief but also experienced powerlessness due to the hierarchical decision‐making structure, where doctors held the ultimate authority over prescribing	I am my patient's advocate and my patient is in pain but if the doctor is not willing to prescribe anything what can I do and especially if it is drug‐seeking behaviour.	Organisational barrier
10.	Rowe (2018), USA Citation search	Nurses experienced restraints on what they could and could not do within the hospital pain management guidelines	I feel angry when my suggestions for pain relief are ignored. I become angry.	Organisational barrier
		Participants experienced a sense of frustration when assessing the psychiatric patient because it was difficult to determine if the patient was in fact experiencing pain or exhibiting attention‐seeking or drug‐seeking behaviours	Knowing if the pain was really real – some residents might be addicted, and might complain of pain to get medications. You really have to cross‐examine them sometimes to see if their complaints are truly valid.	Invisibility of pain
		Nurses felt a deep sense of frustration when assessing psychiatric patients, as it was often challenging to determine whether the patient was genuinely experiencing pain or engaging in attention‐seeking or drug‐seeking behaviours	I have to verify pain every time they state they are in pain with the residents because even if they are drug‐seeking or attention‐seeking, there is the chance that today they may legitimately be in pain, and not crying wolf. I teach my nurses to use things like the Flak's scale and dementia scale.	Trusting the patient
		Providing pain management care	A lot of the younger generation come in already addicted to pain medications so you must be careful there not to put them into withdrawals, but then again, not to give them something that is addicting. So, it is difficult to manage.	Withholding treatment
11.	Riquin et al. (2017), France	Nurses expressed certain difficulties in the evaluation of pain for a patient with schizophrenia particularly distinguishing somatic or psychiatric manifestations	In this context, we treated the pain based on the patient's speech, quickly initiating morphine treatment, given the often very painful bone metastases.	Invisibility of pain
12.	Sullivan et al. (2015), USA Citation search	Clinicians expected persons with schizophrenia would be less adherent to treatment, less able to read and understand educational materials and less capable of managing their health and personal affairs		Diagnostic overshadowing
		Providers were less likely to refer a patient with schizophrenia to a weight reduction program. Other types of referral decisions (for pain management and sleep study) were not influenced by a schizophrenia diagnosis		Withholding treatment

To enhance rigour and trustworthiness, two authors (AA and IN) independently extracted the qualitative findings, including the identified themes and supporting quotations, from the included studies through constant comparison. These extracted materials were subsequently reviewed by the remaining authors (YM, MA, RS) to verify the accuracy, consistency and coherence of the extracted themes.

Theme validation was undertaken through an iterative review process. All five authors examined the alignment between the extracted themes and the supporting participant quotations to ensure that the interpretations accurately reflected the original study findings. Consensus discussions were held to confirm that the quotations appropriately substantiated the identified themes, and any discrepancies were resolved through discussion until agreement was reached among all authors. Data extraction and synthesis were managed using Microsoft Excel. No other specialised qualitative data analysis software was used during this process.

The research team comprised experienced registered nurses from a range of clinical backgrounds, including two mental health nurses and three general nurses. The professional experiences of the reviewers may shape the lens through which the evidence is interpreted. To minimise the influence of individual assumptions or disciplinary perspectives, the processes of data extraction and theme development were iterative, collaborative processes. Reflexive dialogue with all reviewers was undertaken throughout the synthesis process to ensure that interpretations remained grounded in the findings reported in the original studies.

## 4 Results

### 4.1 Study Selection

In total, 1318 potential records were generated from five databases including Medline (*n* = 607), CINAHL (*n* = 255), PsyINFO (*n* = 375), SPORTDiscus (*n* = 36) and Psychology and Behavioural Sciences Collection (*n* = 69). Additional five articles were identified through citation searching. After 376 duplicates were removed, 948 studies were screened for their title and abstract. Based on the inclusion criteria, 41 studies were selected for full‐text review. After full screening, a total of 12 articles were included in this review, and 34 studies were excluded for the reasons listed in Figure 1.

**FIGURE 1:**
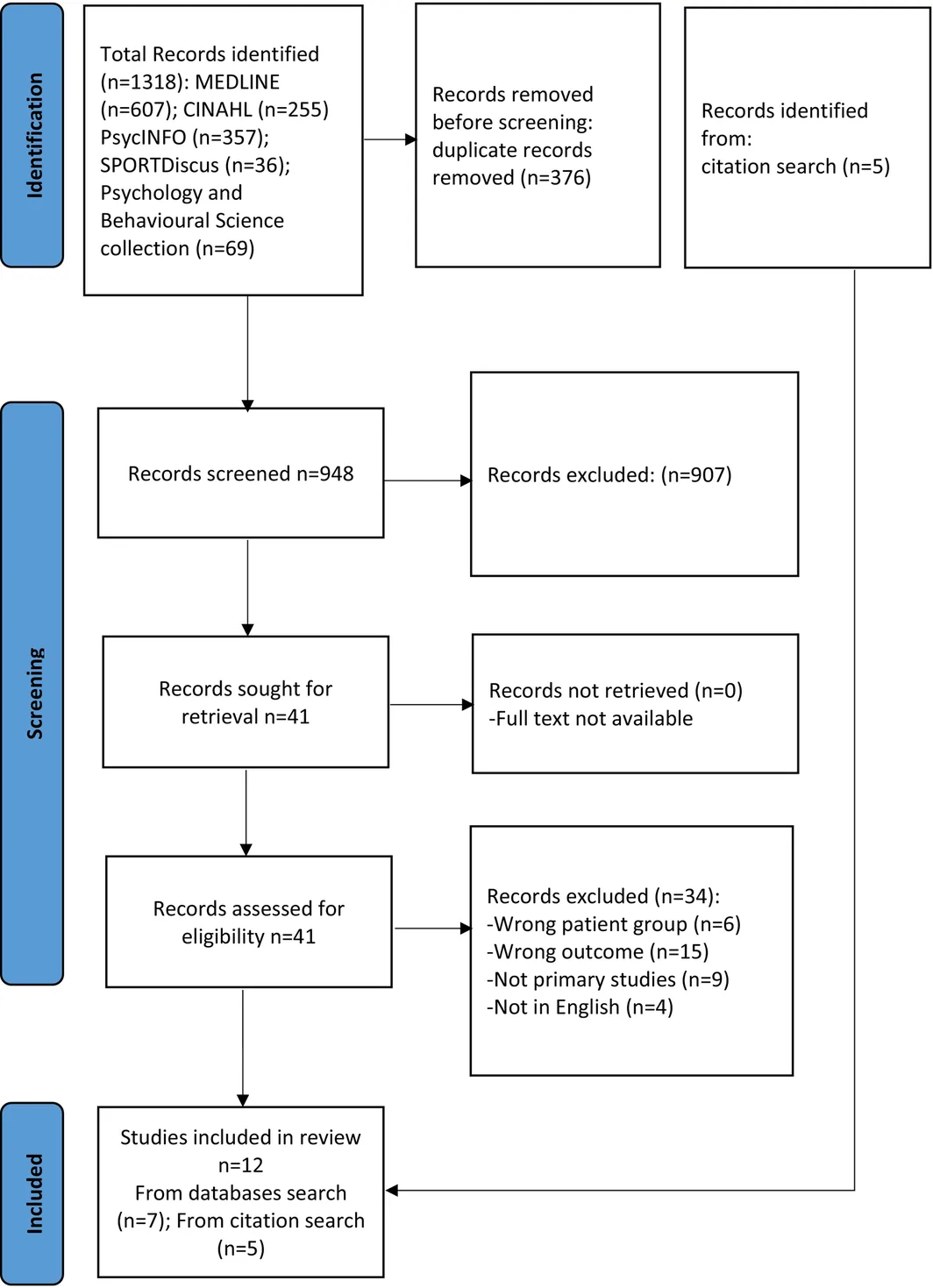
PRISMA flow diagram.

### 4.2 Quality of Included Studies

Eight studies were appraised using the screening criteria for qualitative studies (1.1 to 1.5) and four studies were appraised using the screening criteria for quantitative descriptive studies (4.1 to 4.5). All included qualitative studies used an appropriate qualitative approach and data collection method to answer the research questions. The analysis methods are congruent with findings which are adequately derived from the data and interpretation of findings are sufficiently substantiated with a clear link between data sources, collection, analysis and interpretation.

Similarly, all four quantitative descriptive studies met four out of five criteria (4.1, 4.2, 4.3, 4.5) which included using an appropriate sampling strategy and a sample representative of the target population, applying appropriate measurements and statistical analysis methods to answer the research question. None of the quantitative descriptive studies met the criterion for managing the risk of nonresponse bias due to lack of reporting except one study being a case report (Riquin et al. 2017). The key themes identified from these quantitative descriptive studies were found to support the qualitative study findings and did not appear to change the overall interpretation of the themes.

### 4.3 Characteristics of the Included Studies

The main characteristics of the included studies are illustrated in Table 3 (Data extraction). The evidence base represented studies involving patients with mental health conditions, including substance use disorders. Included articles in this review were published between 2004 and 2020. Articles were published in six different countries. Four studies were conducted in the USA (Morgan 2014, 2016; Sullivan et al. 2015; Rowe 2018), two from the United Kingdom (Morley et al. 2015, 2020), two from Canada (Dewar et al. 2009; Dewar and Osborne 2004) and one from Sweden (Bohm et al. 2019), Israel (Kerner et al. 2013), Norway (Li et al. 2012) and France (Riquin et al. 2017).

All the articles reported the experiences of nurses in assessing and managing pain rather than focusing solely on pain assessment. Nine articles employed qualitative design; three articles were descriptive qualitative (Bohm et al. 2019; Dewar et al. 2009; Dewar and Osborne 2004); grounded theory (Morgan 2014); qualitative phenomenology (Morgan 2016; Morley et al. 2015, 2020; Rowe 2018) and one case study (Riquin et al. 2017). The remaining three studies employed quantitative design; observational retrospective study (Kerner et al. 2013); descriptive cross‐sectional (Li et al. 2012) and cross‐sectional survey (Sullivan et al. 2015).

A total of 80 nurses participated across the qualitative studies, and a total of 587 participants participated in the quantitative studies. Participants were sampled in a variety of settings including acute and long‐term psychiatric units (Dewar et al. 2009; Dewar and Osborne 2004; Riquin et al. 2017; Rowe 2018), medical and surgical specialities (Kerner et al. 2013; Li et al. 2012; Morgan 2014, 2016; Morley et al. 2015; Sullivan et al. 2015) and pre‐hospital emergency services (Bohm et al. 2019).

### 4.4 Main Findings

The aim of this review was to gain an understanding of nurses' experiences of pain management for patients with diagnosed mental health conditions including substance use disorders. This resulted in the identification of a number of barriers and facilitators that influenced the clinical decision‐making process (Figure 2). The key findings are reported below.

**FIGURE 2:**
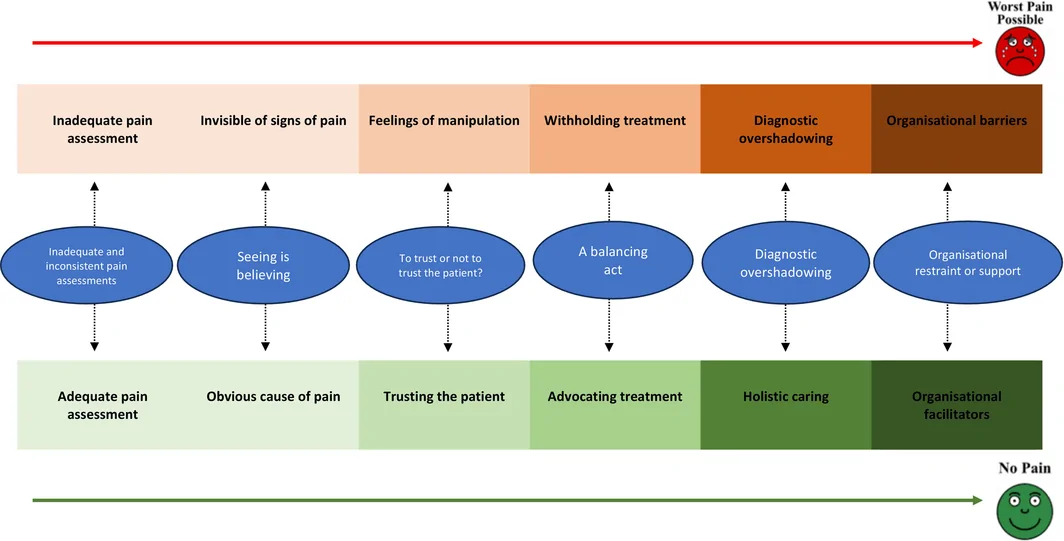
Clinical decision making in pain assessment and management.

#### 4.4.1 Inadequate and Inconsistent Pain Assessments

Nurses use a combination of formal tools and clinical judgement to assess pain, though their approaches vary across settings and patient populations. Tools such as the Numerical Rating Scale (NRS), Visual Analogue Scale (VAS) and behavioural assessments like the Algoplus and FLACC scales are readily available but inconsistently applied in clinical practice (Riquin et al. 2017; Rowe 2018; Kerner et al. 2013; Bohm et al. 2019). Despite the presence of pain assessment tools, many nurses rely heavily on behavioural cues, vital signs and patient appearance to evaluate pain (Bohm et al. 2019; Morley et al. 2015), with over half reporting they have never used a self‐reported or observational pain assessment tool (Li et al. 2012). This inconsistency is particularly problematic when assessing patients with diagnosed mental health conditions, where communication may be impaired and behavioural presentations can be misinterpreted. One nurse highlighted the limitations of using multiple tools in a patient with serious mental illness:

We used the numerical self‐assessment scales (the levels are numbers) or simple verbal (the levels are described by words such as low, moderate, etc.), or the Algoplus hetero‐assessment behavioral scale (scale intended for patients with communication difficulties). The visual analog scale (ruler on which the patient situates his painful sensation) was not used due to a misunderstanding of the instructions. The scales showed discordant results (Riquin et al. 2017, 143).

Moreover, studies also indicate that patients with diagnosed mental health conditions are more likely to have their pain underassessed. Li et al. (2012) found only 23.2% of nurses used pain assessment tools when assessing pain of patients with diagnosed mental health conditions. Kerner et al. (2013) also reported that pain assessment was significantly underperformed in patients whose primary reason for hospitalisation was a mental health condition. This suggests a potential bias, where patients with diagnosed mental health conditions are less likely to receive a thorough assessment of pain using validated pain assessment tools, potentially contributing to inadequate pain management.

#### 4.4.2 Seeing is Believing

The pain experienced by patients with diagnosed mental health conditions was found by Dewar and Osborne (2004) to be perceived and managed differently by healthcare professionals if they were unable to see an obvious medical or surgical cause. Eight of the 12 studies in our review reported that nurses were hesitant to administer pain relief to patients with diagnosed mental health conditions when there are no objective signs of pain, such as abnormal vital signs or visible injuries (Li et al. 2012; Bohm et al. 2019; Dewar et al. 2009; Dewar and Osborne 2004; Morgan 2014; Morley et al. 2020). Bohm et al. (2019) further reported that nurses often felt justified in withholding or minimising analgesia due to the absence of obvious physical symptoms.

Morley et al. (2020) highlighted the challenges of pain assessment in some patients with diagnosed mental health conditions, particularly when there is a discrepancy between self‐reported pain scores and observed physiological responses. This is reflected in a study of pre‐hospital emergency nurses, where one participant stated:

I go very much on body language and parameters. I mean, they're sitting there and talking fluently and saying they have VAS 10 on the pain scale, but they have normal blood pressure and normal pulse and no dilated pupils, so there is nothing physiologically that indicates that they're in pain at all (Bohm et al. 2019, 3).

In contrast, patients with visible and verifiable sources of pain, such as injuries or surgical wounds, receive pain relief promptly and adequately (Bohm et al. 2019; Dewar et al. 2009; Dewar and Osborne 2004). A psychiatric nurse highlighted this disparity, stating:

When they've been on a surgical ward, and they've had a pin put in their leg, and they ask me for Tylenol 3's for that leg pain—I mean, the person has obvious pain. When you're here [psychiatry], it's a little less obvious (Dewar et al. 2009, 298).

The absence of visible physical symptoms in patients with diagnosed mental health conditions often leads to doubt about the legitimacy of their pain, making it difficult for nurses to trust their reports (Dewar et al. 2009; Dewar and Osborne 2004; Rowe 2018). Additionally, nurses frequently experience frustration in distinguishing between genuine pain, attention‐seeking behaviour and drug‐seeking tendencies. These factors influence pain management decisions and may contribute to inadequate treatment for this patient population (Li et al. 2012; Rowe 2018).

#### 4.4.3 To Trust or Not to Trust the Patient?

Nurses reported feelings of manipulation when administering analgesia to patients with diagnosed mental health conditions, particularly those with histories of substance abuse. Studies highlight that some patients may subtly pressure nurses into providing medication, with some opioid‐dependent patients attempting to deceive them when requesting pain relief (Bohm et al. 2019; Dewar et al. 2009; Li et al. 2012). This perception may arise from patients' behaviour when requesting medication. For example, pre‐hospital nurses observed that when patients were overly specific about the medication and its dosage, it raised suspicions that they were seeking drugs (Bohm et al. 2019). Such behaviour sometimes led nurses to make decisions based on the potential escalation of the situation, which could disrupt the therapeutic environment. As one nurse explained:

I want this person settled, I don't want this person totally irate and acting out and directing that towards me. I'd give that med to him (Dewar et al. 2009, 299).

While the administration of analgesia could settle the patient and maintain unit safety, the sense of manipulation and the pressure nurses felt to comply often undermined their morale and sense of professionalism (Dewar et al. 2009). One nurse voiced this frustration:

As a nurse, it just makes me feel that your credibility, not your credibility but your professionalism. You could be the street drug corner guy giving out drugs to this guy, sometimes you feel like a pusher, you feel like the drug dealer, and it makes you feel kind of yucky when you've got this sort of shady guy manipulating you, and it leaves you feeling dirty afterward (Dewar et al. 2009, 299).

Although nurses often struggled to trust patients' complaints of pain especially those with a history of substance abuse, they also expressed a desire to believe their patients and respect their right to treatment (Bohm et al. 2019; Morley et al. 2020; Rowe 2018). This tension between scepticism and trust impacts the nurse–patient relationship, which is central to the administration of pain relief. One participant emphasised the importance of this relationship:

The relationship between patients and nurses is very important. If you think about it, they've left their homes, or if they have homes or not, they're here, and to me nursing, like I tell other nurses, somebody's life is in your hands. They are trusting you, and their life is in your hands. So if you don't look at pain, and then you don't treat them, you know, it's like you are contributing to their condemnation. So long as the person is here and we've taken a vow to care for the patient, I think we should do it with all our heart (Morgan 2014, 172).

Despite this commitment to patient care, nurses often find themselves confronted with scepticism when providing pain‐relieving medications to patients with a history of substance abuse. As one nurse explained:

If she rates her pain as high on a scale of 1 to 10, I would still give it to her. I would believe her, but just having a previous addiction, it would still make me sceptical (Dewar et al. 2009, 299).

#### 4.4.4 A Balancing Act

Nurses often face the difficult task of balancing effective pain management with the perception of contributing to substance dependency (Bohm et al. 2019; Dewar et al. 2009; Morley et al. 2020). While acting in accordance with beneficence, some nurses may hesitate to administer opioids due to fears of exacerbating patients' substance addiction (Morley et al. 2020). This dilemma is captured by one nurse's reflection:

I don't know how to phrase this in a way that is appropriate, but I think there is always that worry that perhaps giving opiates to somebody who is addicted to opiates is not a good idea, but sometimes that is what they need (Morley et al. 2020, 202).

The fear of triggering opioid dependency often results in suboptimal pain management, with nurses opting for non‐opioid medications or alternative pain‐relief strategies, even in cases involving severe injuries (Dewar et al. 2009; Morley et al. 2020). Additionally, the way nurses assess a patient's presentation can influence their judgement on whether to withhold pain relief. For example, one nurse describes the impact of such judgement:

We've ended up in situations where we meet people who, well, fake an emergency just to get hold of drugs. Of course, they never get given drugs (Bohm et al. 2019, 3).

Despite instances where pain relief was withheld, some nurses demonstrated strong advocacy for patients they believed genuinely needed pain relief however this was not taken seriously by the medical teams (Morgan 2014, 2016; Morley et al. 2020; Rowe 2018). Many expressed a desire to have done more to advocate for their patients' needs. One nurse shared a poignant example:

The patient was just writhing in pain, and the orthopedic surgeon and anaesthesiologist were standing there saying, ‘I don't believe you.’ And then I looked at them and said, ‘Wow, I sincerely hope that someday you're lying in a bed, and the doctor and anaesthesiologist come to you while you're in intractable pain and say’, I don't believe you (Morgan 2014, 171).

Some nurses expressed feeling powerlessness when they felt they did not advocate strongly enough for their patients (Morgan 2016; Morley et al. 2015). As one nurse reflected:

I didn't take a strong enough stance with the (medical) providers who didn't want her to get methadone. It is something I always regretted and really learned from (Morgan and Wells 2016, 37).

#### 4.4.5 Diagnostic Overshadowing

Diagnostic overshadowing is evident in the management of pain for patients with mental health conditions (Dewar and Osborne 2004; Kerner et al. 2013; Morley et al. 2015; Sullivan et al. 2015). Despite pain being assessed quickly upon admission, once a ‘psychiatric label’ was applied, it often becomes a secondary priority (Dewar and Osborne 2004). Studies have shown that patients with diagnosed mental health conditions receive fewer pain assessments compared to those with medical health conditions (Kerner et al. 2013). Some nurses raised concerns about medical management, which prioritises addressing mental health conditions, such as substance abuse, over the alleviation of pain (Morley et al. 2015). As a result, pain may not be adequately assessed or treated, leading to prolonged hospital stays and increased suffering (Dewar and Osborne 2004). A nurse recalled

I remember one of the juniors [doctors] in particular said, ‘I am so glad you have said that. Our consultant just thinks we can get him fixed’ and I said yes, well they will, that is their mindset but this is our reality (Morley et al. 2015, 707).

Nurses recognised the importance of providing pain relief and treating patient holistically (Bohm et al. 2019; Morgan 2016; Rowe 2018). This is particular evident in the care of patients with serious mental health issues during end‐of‐life care where‐in nurses strive to maintain a positive attitude (Morgan 2016). Pre‐hospital emergency nurses expressed the importance of other measures such as building trust and providing security and reassurance for the patient in a calm encounter.

#### 4.4.6 Organisational Restraint or Support

A key finding was the organisational factors that either facilitated or hindered effective pain management for patients with diagnosed mental health conditions. Nurses identified organisational barriers that restrained their ability to provide effective pain management included rigid hierarchies that disempowered nurses thereby limiting their ability to advocate for the patient, nurse‐to‐nurse power dynamics that promoted cautious decision‐making, and systemic issues such as low staffing, high patient acuity, limited access to prescribers and staff shortages (Dewar et al. 2009; Morgan 2014; Rowe 2018). Financial pressures, lack of protected time for education and policies focused on discharging patients as soon as possible further entrenched a culture where nurses felt constrained by institutional structures (Morley et al. 2020). Additionally, nurses faced restrictions when advocating for pain management due to limiting hospital guidelines (Rowe 2018). One nurse expressed frustration at having their pain relief suggestions ignored:

I feel angry when my suggestions for pain relief are ignored. I become angry (Rowe 2018, 67).

Organisational facilitators included access to peer and professional support, which enhanced nurses' decision‐making confidence and reduced isolation (Bohm et al. 2019). Leadership by experienced nurses was considered a facilitator as they tend to foster a calm, therapeutic environment that would benefit both staff and patients (Dewar et al. 2009). Clinical exposure to pain and substance use disorders, along with emotional and professional support with complex cases such as end‐of‐life care, further strengthened nurses' capacity to manage pain effectively (Morgan and Wells 2016). One nurse in the Morgan and Wells (2016) study highlighted the need for integrated educational programs to explore mental illness and addiction while emphasising that pain management education should also include the understanding that prescribing opioid analgesics does not necessarily lead to addiction:

I think they have to look at it and have an integrated program of not only serious mental illness, but also weave in addiction because the two in my opinion, it can't be one without the other. I think there is also a need to educate people in dealing with pain management and that number one, just because narcotics are prescribed you're not going to make everyone an addict (Morgan 2016, 38).

## 5 Discussion

This mixed‐methods systematic review aimed to synthesise the existing evidence on nurses' pain assessment and management practices for patients with diagnosed mental health conditions including substance use disorders. The findings identified six interrelated themes that collectively shape nurses' approaches to assessing and managing pain, with stigma continuing to affect the health experiences of this vulnerable population. These themes include (1) Inadequate and inconsistent pain assessments, (2) Seeing is believing, (3) To trust or not to trust the patient? (4) A balancing act, (5) Diagnostic overshadowing and (6) Organisational restraint or support.

Despite growing recognition of mental health conditions and substance use disorders as having a significant health impact, stigma remains pervasive and continues to shape how individuals are perceived and treated in healthcare settings (Kågström et al. 2025). Stigma influences nurses' pain assessment and management practices and was evident in this review through the themes such as ‘Seeing is believing’ and *‘*To trust or not to trust the patients’ leading to reduced analgesic administration and, in some cases withholding pain relief altogether (Bohm et al. 2019; Dewar et al. 2009; Li et al. 2012). These practices were more apparent for patients with a history of substance use disorders, whose pain expressions were more frequently met with suspicion and scepticism.

Nurses also described patients who clearly articulated request for specific analgesics as ‘manipulative’ or ‘drug‐seeking’, reflecting deeply embedded societal stigma (Bohm et al. 2019; Dewar et al. 2009; Morley et al. 2020). This is particularly concerning given that approximately 46% of people with a diagnosed mental health condition also have a co‐occurring substance use disorder (Sunderland et al. 2025). Such stigmatisation may restrict access to pain relief, intensify pain experiences and worsen mental health outcomes (Zhang et al. 2023).

While the preceding findings highlight the role of individual perspectives in shaping nurses' pain management practices, evidence suggests that these experiences are also influenced by broader organisational and institutional pressures (Joniak‐Grant et al. 2023). At an organisational level, stigma is reinforced through organisational structures and processes, particularly in relation to opioid prescribing. For example, restrictive hospital guidelines could limit nurses' ability to advocate for pain relief (Rowe 2018) and affect their ability to advocate for patients which can be further constrained when doctors are reluctant to prescribe the analgesics (Morgan 2016; Morley et al. 2015). These findings align with our review theme on ‘Organisational restraint or support’ indicating that within these hierarchical decision‐making structures, nurses' suggestions for pain relief may be overlooked or disregarded, leading to frustration and anger among nurses when their clinical assessments are not acknowledged (Rowe 2018).

Similarly, another form of stigma evident in this review was ‘*Diagnostic overshadowing*’ (Dewar and Osborne 2004; Kerner et al. 2013; Morley et al. 2015; Sullivan et al. 2015). Nurses reported that once a mental health diagnosis was established, patients' reports of pain were often disregarded or considered a lower priority, leading to reduced pain assessment and under‐treatment (Dewar and Osborne 2004; Kerner et al. 2013). While nurses recognised this as problematic, they often felt disempowered by the rigid hierarchies that limited their ability to act and advocate for patient's pain relief (Dewar et al. 2009; Morgan 2014; Rowe 2018).

Besides the interaction between individual and structural stigma that shapes nurses' clinical decision‐making processes in pain management for patients with diagnosed mental health conditions, the review has found nurses often rely heavily on ‘visible’ signs of pain and at the expense of patients' subjective reports (Li et al. 2012; Bohm et al. 2019; Dewar et al. 2009; Dewar and Osborne 2004; Morgan 2014; Morley et al. 2020). This finding aligns well with Tanner's Clinical Judgement Model which emphasised the phases of noticing and interpreting and subjective pain reports often carry less weight (Tanner 2006). While noticing observable cues is a critical component of clinical judgement, this is insufficient for patients with diagnosed mental health conditions, who may mask or underreport pain due to fear of judgement, prior stigmatisation, or pain invalidation (Boring et al. 2024). Consequently, the absence of overt pain behaviours or behaviours that are attributed to the mental health condition or substance use disorders may be misinterpreted as an absence of pain, increasing the risk of under‐assessment and inadequate analgesic management (Li et al. 2012; Bohm et al. 2019; Dewar et al. 2009; Dewar and Osborne 2004; Morgan 2014; Morley et al. 2020).

These patterns of clinical judgement further reflect a broader biomedical orientation to pain, in which credibility is closely tied to observable signs and verifiable pathology. Pain has historically been dominated by a *‘biomedical model’*, where legitimacy is conferred through visible pathology rather than patients' subjective experiences (Pianese and Bordoni 2025). Our review reinforces this, demonstrating that nurses often struggled to recognise pain in the absence of clear somatic markers, indicating that non‐physical dimensions of pain are frequently under‐recognised (Bohm et al. 2019; Dewar et al. 2009; Dewar and Osborne 2004; Morgan 2014; Morley et al. 2020).

Moreover, the documentation process in clinical practice also reinforces the prioritisation of quantifiable indicators over subjective reports. For example, patients' subjective pain experiences are often transformed into numerical scores using pain assessment tools embedded in routine practice to orient clinical attention toward observable signs, thus marginalising the multidimensional nature of pain (Janerka et al. 2026). Consequently, the interpretation phase of clinical judgement may be compromised, as decisions are shaped by what is initially observed, increasing the likelihood that pain is minimised, misattributed to the mental health diagnosis, or not recognised as requiring treatment.

To address these limitations, ‘Total Pain Theory’ (Saunders 1964) offers a multidimensional framework for understanding pain by encompassing physical, psychological, social, emotional and spiritual domains rather than solely physiological symptoms (Mehta and Chan 2008). It provides a practical guide for nurses to navigate these complexities when caring for patients with diagnosed mental health conditions, whose emotional distress, social challenges and prior experiences of stigma can mask or alter the presentation of pain (Boring et al. 2024). Nurses are encouraged to attend to visible physiological signs and subjective reports, emotional expressions and social context to develop a holistic and person‐centred intervention for pain management. By recognising the interconnected dimensions of pain experience through Total Pain Theory would also encourage a multidisciplinary approach, ensuring healthcare professionals collaborate to address patients' complex pain needs (Corman et al. 2025).

This review highlights the ongoing challenges in providing adequate analgesia for patients with diagnosed mental health conditions, including substance use disorders. It has highlighted the complex interplay between organisational expectations, the dominant biomedical model, the focus on objective rather than subjective pain experiences and the ethical and professional dilemmas nurses face when advocating for appropriate management for this vulnerable group. A key strength of the review was its integration of qualitative and quantitative evidence, which provided a comprehensive understanding of the barriers and facilitators to effective pain assessment and management. It also illuminated the unique challenges faced by a vulnerable population, particularly when navigating subjective pain reports in the context of psychiatric comorbidities and societal stigma.

Despite these strengths, several limitations should be acknowledged. The review predominantly included English‐language studies, which may introduce language bias and exclude relevant research from non‐English‐speaking contexts. The evidence is largely drawn from Western countries, limiting the generalisability of findings to other cultural or healthcare contexts. Furthermore, the included studies varied in methodological quality. Although a formal sensitivity analysis was not undertaken, it is possible that findings from studies with methodological limitations may have influenced the synthesis and interpretation of themes. As such, the conclusions of this review should be interpreted with appropriate caution, particularly where themes were supported by a limited number of studies.

### 5.1 Recommendations

The findings of this review suggest that healthcare institutions should strengthen training for nurses and other healthcare professionals in pain assessment, particularly for people with mental health conditions including substance use disorders. Nurses, especially those working in general health settings, need support and skill development to engage in challenging conversations with patients experiencing substance use disorders, with prescribers and other team members. Current literature shows that general nurses have difficulties initiating deeper and difficult conversations with patients with mental health conditions (Ngune et al. 2025).

Education should emphasise the integration of clinical judgement with evidence‐based pain assessment tools to support objective and consistent pain evaluations, particularly for patients with mental health conditions and substance use disorders. The aim should be enhancing nurses' understanding of how individuals with mental health conditions and substance use disorders experience pain, including how responsive behaviours may affect pain expression and assessment. This may reduce misinterpretation of pain symptoms as purely psychiatric and help reduce diagnostic overshadowing. Further education is also needed to develop understanding of the complex relationship between chronic pain and opioid dependence, including the management of hyperalgesia and tolerance. There should be a focus on evidence‐based approaches to managing acute pain in individuals with a history of opioid dependence.

Awareness of the role of trauma for people experiencing mental health conditions and substance use disorders is also needed to holistically care for this group. Pain management strategies should include trauma‐informed principles of trust, collaboration and empowerment, recognising the high prevalence of trauma in individuals in this group. This approach will enhance trust, reduce re‐traumatisation and support patient‐centred care.

## 6 Conclusion

This review highlights the ongoing challenges nurses encounter in assessing and managing pain in patients with diagnosed mental health conditions including substance use disorders. There continues to be a need for enhanced education and institutional support to strengthen nurses' capacity for effective pain assessment and management for this group. Stigma both structural and interpersonal can shape clinical decision‐making, often leading to the under‐assessment and inadequate treatment of pain. Addressing biases through education and supportive policy is essential. Equipping nurses with the skills to integrate clinical judgement with validated assessment tools will help ensure consistent, evidence‐based and person‐centred pain care for this often‐marginalised group.

## Funding

The authors have nothing to report.

## Conflicts of Interest

The authors declare no conflicts of interest.

## Data Availability

Data sharing not applicable to this article as no datasets were generated or analysed during the current study.
